# Biofilm vertical growth dynamics are captured by an active fluid framework

**DOI:** 10.1088/1478-3975/ade928

**Published:** 2025-07-11

**Authors:** Raymond Copeland, Peter J Yunker

**Affiliations:** School of Physics, Georgia Institute of Technology, Atlanta, GA, United States of America

**Keywords:** biofilms, microbes, active fluid model, vertical growth

## Abstract

Bacterial biofilms, surface-attached microbial communities, grow horizontally across surfaces and vertically above them. Although a simple heuristic model for vertical growth was experimentally shown to accurately describe the behavior of diverse microbial species, the biophysical implications and theoretical basis for this empirical model were unclear. Here, we demonstrate that this heuristic model emerges naturally from fundamental principles of active fluid dynamics. By analytically deriving solutions for an active fluid model of vertical biofilm growth, we show that the governing equations reduce to the same form as the empirical model in both early- and late-stage growth regimes. Our analysis reveals that cell death and decay rates likely play key roles in determining the characteristic parameters of vertical growth. The active fluid model produces a single, simple equation governing growth at all heights that is surprisingly simpler than the heuristic model. With this theoretical basis, we explain why the vertical growth rate reaches a maximum at a height greater than the previously identified characteristic length scale. These results provide a theoretical foundation for a simple mathematical model of vertical growth, enabling deeper understanding of how biological and biophysical factors interact during biofilm development.

## 1. Introduction

Biofilms are surface-attached, crowded communities of microbes that can grow in two directions: horizontally across the surface or vertically above the surface [1–3]. While horizontal growth and range expansion has been more extensively studied [4–7], a growing body of evidence suggests that vertical growth is a crucial aspect of bacterial behavior [8–12]. It has even been shown that vertical growth can impact horizontal growth, implying that colony expansion can only be fully understood by considering both phenomena [13]. Additionally, in some environments, confinement can prevent horizontal growth while allowing vertical growth to continue. Therefore, understanding biofilm growth requires a comprehensive examination of vertical growth dynamics.

Our understanding of vertical growth has been limited in part by its complexity. Nutrients diffuse into the colony through its interfaces, and then are uptaken by microbes. Thus, in a single vertical direction, bacteria experience many different microenvironments [14, 15]. There are many approaches to modeling such systems, cellular automata-based models [16–18], individual based models [4, 19–21], or to treat the microbes as an active fluid. Models can even mix aspects of these approaches [22, 23]. In active fluid models, microbes are treated as growing and energy-consuming particles characterized by viscous interactions, allowing for analytical modeling of biofilms based on fluid principles [24, 25]. Prior research has modeled the expansion of biofilms via nutrient diffusion, uptake, and growth as an active fluid [26–32].

**Figure 1.**
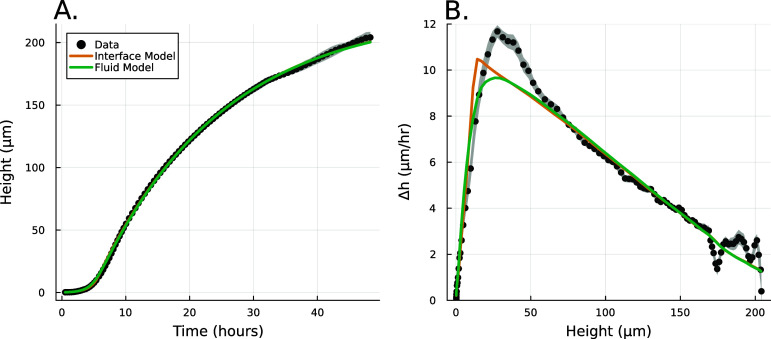
(A) Experimental data from Bravo *et al* [15] depicting the growth of A. veronii colony height over time, measured using an interferometer. The interface model was applied using equation (1) and the active fluid model using equation (27). Fit parameters for the active fluid model are $\alpha = 0.86 \, \text{h}^{-1}$, $\beta = 0.051 \, \text{h}^{-1}$, and $L = 13.17 \, \mu m$; for the interface model, $\alpha = 0.82 \, \text{h}^{-1}$, $\beta = 0.050 \, \text{h}^{-1}$, and $L = 13.86 \, \mu m$.(B) This panel shows the slope of the height over time curve as a function of height, with experimental data again sourced from Bravo *et al* [15]. The active fluid model is represented by the green line and the interface model by the orange line, as in panel A.

A recent paper, Bravo *et al* [15], proposed and experimentally validated a simple heuristic model which we will refer to as the interface model (see figure 1):

\begin{align*} \frac{\mathrm{d}h}{\mathrm{d}t} = \begin{cases} \left(\alpha - \beta\right) h &amp; h\unicode{x2A7D} L \\ \alpha L - \beta h &amp; h \unicode{x2A7E} L \end{cases}\end{align*}

While this model captured the empirical behavior of nine different species, including prokaryotes and eukaryotes, gram negative and gram positive bacteria, and cells with a wide range of shapes and sizes, it was proposed based on empirical data, and justified term-by-term, rather than derived from fundamental dynamics. Thus, the underlying biophysics behind vertical growth remains unclear.

Further, the interface model was unable to explain multiple empirical observations. In particular, the vertical growth rate consistently reached a maximum at biofilm heights greater than the characteristic growing region of length *L*. This effect has also been observed at the edge of growing colonies [13]. However, without a pure theoretical foundation on which to develop hypotheses, it is challenging to investigate the deviations between the model and empirical observations.

Here, we show that the interface model from Bravo *et al* [15], arises directly from a commonly used active fluid model of biofilm growth. We derive the interface model from analytical expressions of the active fluid model, and then we fit the experimental data from Bravo, *et al* to both the active fluid model and the interface model, showing that the heuristic model is nearly as good (see figure 1). Moreover, we identify that the discrepancies between the interface model and the observed maximum growth rates are effectively captured by the active fluid model. Thus, we conclude that vertical growth can be accurately modeled using the simpler heuristic framework proposed by Bravo *et al* [15], with minimal deviation.

## 2. Active fluid model

Here, we develop a one-dimensional model for vertical biofilm growth, with the goal of producing a model for the height over time in alignment with experimental observations. We initiate this process by deriving a model for a growing ‘bio-fluid’ based on the principles of active fluid dynamics. The derivation here closely follows the methodologies established in previous works [22, 27, 28, 30, 33]. We consider a biofilm, consisting of a single species of cells, growing vertically upward. We let $m(z, t)$ be the cell mass density at a distance, *z*, above the base of the biofilm at time *t*. We also consider the mass concentration of a generic diffusible resource, $R(z, t)$, (e.g. a nutrient) which is consumed by the cells with rate $f(m,R)$. The mass continuity equation is then:

\begin{align*} \frac{\partial m}{\partial t}+\frac{\partial}{\partial z}\left(u m\right) = g\end{align*}

where *u* is the velocity field of the system and *g* is the growth function; this will be a function the resources *R*. Note, diffusion of the bio-fluids is assumed to be negligible [24]. The resources diffuse, move under the same velocity field as the bio-fluids, and are uptaken by each mass species in the environment:

\begin{align*} \frac{\partial R}{\partial t} = D_R\frac{\partial^2 R}{\partial z^2}- \frac{\partial}{\partial z}\left(uR\right)-f\left(m,R\right)\end{align*}

where *D_R_* is the diffusion coefficient and there is some functional form of consumption $f(m,R)$ for the system’s mass species. The resources themselves are small molecules and thus we do not model them as having volume in the same way as the bio-fluid.

As the bio-fluid can be treated as incompressible (they are mostly made of water [34]), we can re-frame the equations away from mass density, instead replacing them with volume fraction $N = m/\rho$ where *ρ* is the specific density of the cells themselves. This leaves *N* as the volume occupied by our species divided by the total volume at a given position. We also replace the growth function with volume growth function $g^* = g/\rho$:

\begin{align*} \frac{\partial N}{\partial t}+\frac{\partial}{\partial z}\left(uN\right) = g^*.\end{align*}

Note this one dimensional, incompressible, single species approach also allows for a ‘no voids’ constraint, meaning that at every position within the biofilm, the volume fraction of ‘bio-fluid’ must fill all available space:

\begin{align*} N\left(z,t\right) = 1.\end{align*}

We can now take the volume density continuity equation (4) and apply the constraint equation (5) to arrive at an expression relating the velocity field and growth:

\begin{align*} \frac{\partial u}{\partial z} = g^*.\end{align*}

It is next assumed that this bio-fluid can be treated as homogeneous, viscous, and incompressible with constant density flowing through a porous environment, satisfying Darcy’s law; this allows us to model the global velocity field with a scalar potential, i.e. the pressure field *p*:

\begin{align*} u = -\lambda \frac{\partial p}{\partial z}.\end{align*}

Finally, the growth function must be defined. The terms in the growth function indicate the increase or decrease of the bio-fluid volume; hence, a positive term signifies volume creation, while a negative term indicates volume loss. Our species grows in proportion to both its concentration and the availability of resources at a specific location (*R*; it also experiences decay at rate *β*. Thus, our growth function is:

\begin{align*} g^* = N\left(cR-\beta\right) = \alpha\frac{R}{R^*} - \beta\end{align*}

where equation (5) allows us to replace *N* with 1 (equation (8)). Note, the decay rate *β* signifies the rate at which the bio-fluid volume decreases, not the disappearance of the mass itself, but rather the reduction in volume occupied by the bio-mass. We assume that cells degrade on a timescale that is short compared to the timescale of growth; hence degradation is treated as instantaneous. Additionally, while alternative common growth functional forms related to resource consumption, such as the Monod equation, could certainly be utilized, the form we have adopted facilitates the solution of the system. The use of an unbounded maximum growth rate as *R* increases could potentially introduce complications; however, we will implement a Dirichlet boundary condition on the resources at $R^*$ on the bottom layer of the colony (see equation (11)). $R^*$ is then a prescribed concentration of resources at the bottom layer, stabilizing *R* to be at most $R^*$ (so long as the colony does not drop suddenly in height leading to a compressed R level). Thus, while growth is typically defined explicitly by a predetermined maximum growth rate *α*, we can still impose such a maximum by aligning it with realistic values through the use of constant *c* , such that $\alpha = cR^*$. Thus, we replace *c* with *α*.

Now, we must define the resource consumption term which is now a function of *N* not *m* as we re-framed away from mass density. As seen above, the rate at which resources are consumed and made into the bio-fluid is controlled by the constant *c*, which has units of one over mass-density time. This leads to the following consumption term where *ε* is a yield constant, with units of volume fraction of bio-fluid produced per concentration of resources consumed [35]:

\begin{align*} f\left(N,R\right) = \frac{1}{\epsilon}cNR = \frac{1}{\epsilon}\alpha \frac{R}{R^*}.\end{align*}

To continue towards our goal of generating an expression for the height of the colony over time, we next define the rate at which the height changes over time. As it is solely a function of the velocity *u* at the top of the colony, we can write it as such:

\begin{align*} \frac{\partial h}{\partial t} = u\left(z = h,t\right).\end{align*}

We now define our boundary conditions. The gradient of pressure must be zero at *z* = 0 (which imposes that the biomass velocity field must be zero at *z* = 0, thus the biomass cannot flow into the ground). We imposed a Dirichlet boundary (*p* = 0) at the top of the colony (*z* = *h*) in line with previous work [24, 33] as pressure is only unique up to an additive constant. For the resources, we assume that the agar underneath the colony acts as source (at *z* = 0) such that its concentration is constant (see equation (11)), and the gradient of *R* is zero at the top of the colony (see equation (12)). Thus, resources flow freely from the bottom of the colony, but they cannot flux out of the top of it:

\begin{align*} R\left(z = 0,t\right) = R^*\end{align*}

\begin{align*} \frac{\partial R}{\partial z}\left(z = h,t\right) = 0\end{align*}

\begin{align*} \frac{\partial p}{\partial z}\left(z = 0,t\right) = 0\end{align*}

\begin{align*} p\left(z = h,t\right) = 0.\end{align*}

At this point we note that we have not defined any initial conditions for our system. This is intentional as our goal is to find an equation for the height of the colony over time which we will then fit to the experimental data from [15] in the same manor as the interface model.

## 3. Derivation of heuristic model

We now derive a solution for the height of the colony over time from the active fluid model from section 2. To do so, we must find the velocity field at the top of the colony (equation (10)), which means we must solve for the pressure gradient (equation (7)), which means we must know the solution of the growth functions (equation (6)), which means we must find the solution for *R* (equations (17) and (9)). This expression has a time dependency and the unknown velocity field *u*. To address this, we nondimensionalise the system with the following scalings:

\begin{align*} \begin{aligned} t &amp; = \hat{T}_{\text{adv}} t^{^{\prime}}, &amp; R &amp; = R^* R^{^{\prime}}, &amp; h &amp; = h_0 h^{^{\prime}} \\ u &amp; = \frac{h_0}{\hat{T}_{\text{adv}}} u^{^{\prime}}, &amp; \epsilon &amp; = \frac{1}{R^*}\epsilon^{^{\prime}}, &amp; z &amp; = h_0\tilde{z}. \end{aligned}\end{align*}

Where the advection time is based on cell-movement and thus the growth rate ($\hat{T}_{\text{adv}} = 1/\alpha$), and *h*_0_ is the initial height of the colony (≈ 1 cell length). This also allows the introduction of the following dimensionless parameters

\begin{align*} \begin{aligned} P_e &amp; = \frac{\hat{T}_{\text{diff}}}{\hat{T}_{\text{adv}}} = \frac{\alpha h_0^2}{D_R}\\ \alpha^{^{\prime}} &amp; = \alpha \frac{h_0^2}{D_R} \end{aligned}\end{align*}

where $\alpha^{^{\prime}}$ is the growth rate scaled by the diffusion time and *P_e_* is the Péclet number (the ratio of diffusion time to advection time) This leaves us with the following expression for the time dependent resource equation:

\begin{align*} \frac{\partial R^{^{\prime}}}{\partial t^{^{\prime}}}+ \frac{\partial}{\partial \tilde{z}}\left(u^{^{\prime}}R^{^{\prime}}\right) = \frac{1}{P_e}\frac{\partial^2 R^{^{\prime}}}{\partial \tilde{z}^2}-\frac{\alpha^{^{\prime}}}{\epsilon^{^{\prime}} P_e}R^{^{\prime}}.\end{align*}

Using values from [15] *P_e_* is $\approx10^{-4}$, implying that diffusion is significantly faster than advection and growth in our system. This ‘rapid’ diffusion of resources is an important characteristic of such systems, and it allows us to neglect the advection and time dependent terms from (17). This leaves us with the quasic static approximation in which we return to dimensional units:

\begin{align*} \frac{\partial^2 R}{\partial z^2}\approx\frac{\alpha}{\epsilon R^*}R.\end{align*}

And the general solution:

\begin{align*} R\left(z\right)\approx C_1e^{z/L}+C_2e^{-z/L}.\end{align*}

Using the boundary conditions defined in equations (11) and (12), we arrive at the following expressions for our unknown constants. We find a characteristic length scale *L* (equation (20)); it is proportional to the diffusion of resources, their concentration, and inversely to the growth rate. The functional form of this length scale is similar to characteristic lengths in horizontal growth models [36]. It will become clear later that this *L* is the same *L* as in the interface model:

\begin{align*} L = \sqrt{\frac{D_R\epsilon R^*}{\alpha}}\end{align*}

\begin{align*} C_1 = \frac{R^*}{e^{2h/L}+1}\end{align*}

\begin{align*} C_2 = \frac{R^*}{e^{-2h/L}+1}.\end{align*}

**Figure 2.**
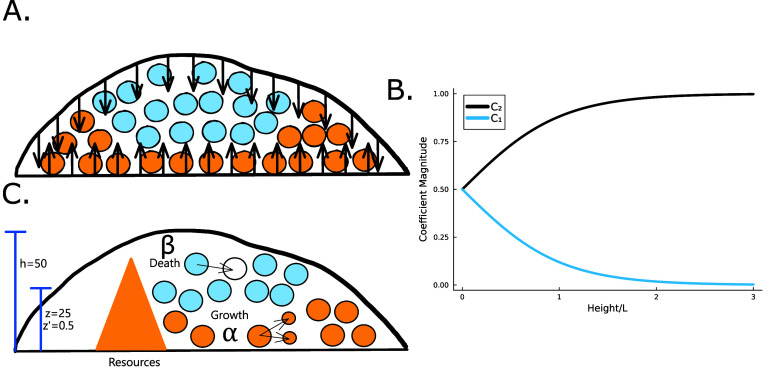
(A) When the total height of the colony is small, the no-flux top surface of the biofilm allows for more growth (orange cells) than when the colony is large, and the cells beyond *L* can no longer grow (blue cells). (B) The magnitude of the coefficient *C*_1_ is plotted over height of the colony where $C1$ is the coefficient of the exponentially growing term in equation (19) while *C*_2_ is the coefficient of the exponentially decaying term. Thus, we see that at small heights *C*_1_ has some impact, while *C*_1_ has little-to-no impact at larger heights. (C) A schematic of our system outlining the coordinate transformation to *z*′, growth rate *α*, death rate *β*, and diffusion of nutrients.

Thus, as *h* grows, the contribution of the positive exponential term decays; however it is significant in early times when *h* is low, see figure 2(B). The combination of these terms results in higher concentrations of resources when the height is low; this is illustrated in figure 2(A) and show in figure 5(A). It is now more convenient to proceed with a hyperbolic solution rather than exponential; thus, our resource curve is the following:

\begin{align*} R \approx R^*\frac{\cosh\left(\frac{1}{L}\left(z-h\right)\right)}{\cosh\left(h/L\right)}.\end{align*}

It is further convenient to undergo a coordinate transformation according to equation (24). This does not change any of the results we already have derived so far, but it will change our derivative functions going forward according to the equations below. This transformation allows us to rigorously define the domain $z^{^{\prime}} \in (0,1)$. Similar coordinate transformations were used in [27, 28, 33]:

\begin{align*} \begin{aligned} z^{^{\prime}} &amp; = \frac{z}{h}, \\ t^{^{\prime}} &amp; = t, \\ \frac{\partial}{\partial z} &amp; = \frac{1}{h} \frac{\partial}{\partial z^{^{\prime}}}, \\ \frac{\partial^2}{\partial z^2} &amp; = \frac{1}{h^2} \frac{\partial^2}{\partial z^{^{\prime} 2}}, \\ \frac{\partial}{\partial t} &amp; = \frac{\partial}{\partial t^{^{\prime}}} - \frac{z^{^{\prime}}}{h} \frac{\partial h}{\partial t^{^{\prime}}} \frac{\partial}{\partial z^{^{\prime}}}. \end{aligned}\end{align*}

With this coordinate transformation we can express the growth of our bio-fluid on a defined domain, see equation (25):

\begin{align*} g_1^*\left(z^{^{\prime}}\right) = \alpha\left(\frac{\cosh\left(\frac{h}{L}\left(z^{^{\prime}}-1\right)\right)}{\cosh\left(h/L\right)} \right)- \beta.\end{align*}

Now we can combine our expression for the growth of the bio-fluid with our expressions for velocity and pressure (equations (6) and (7)) which yields the following expression for the second derivative of pressure (see equation (26)). The extra factor of *h*^2^ appears from the coordinate transformation:

\begin{align*} \frac{\partial^2 p\left(z^{^{\prime}}\right)}{\partial z^{^{\prime} 2}} = \frac{-h^2}{\lambda}\left(\alpha\frac{\cosh\left(\frac{h}{L}\left(z^{^{\prime}}-1\right)\right)}{\cosh\left(h/L\right)}-\beta\right).\end{align*}

As the pressure is in not a function of itself and only spatially defined by *z*′, we can integrate across the whole domain, and then use the boundary conditions for pressure to find the pressure gradient at the top of the colony. We then use this pressure gradient and equation (7) in the modified coordinates (which cancels out another factor of h) to find the velocity at the top of the colony. Finally, with equation (10), we have arrived at an expression for the rate that the height changes over time:

\begin{align*} \frac{\partial h}{\partial t^{^{\prime}}} = \alpha L \tanh\left(h/L\right) - \beta h.\end{align*}

Thus, we recover the interface model described in Bravo *et al*. The active growth regime of length *L* is the characteristic length of diffusing resources as suggested in the study. At $h \gg L$, we are left with:

\begin{align*} \frac{\partial h}{\partial t^{^{\prime}}} = \alpha L-\beta h\end{align*}

and at $h \ll L$:

\begin{align*} \frac{\partial h}{\partial t^{^{\prime}}} = \alpha h-\beta h.\end{align*}

Using the expression in (27) allows us to find the height at which the growth rate is maximized by taking the derivative with respect to *h* and setting the expression to zero:

\begin{align*} h^* = L \operatorname{arcsech} \left(\sqrt{\frac{\beta}{\alpha}}\right).\end{align*}

## 4. Comparison between experiments and approximate numerical predictions

**Figure 3.**
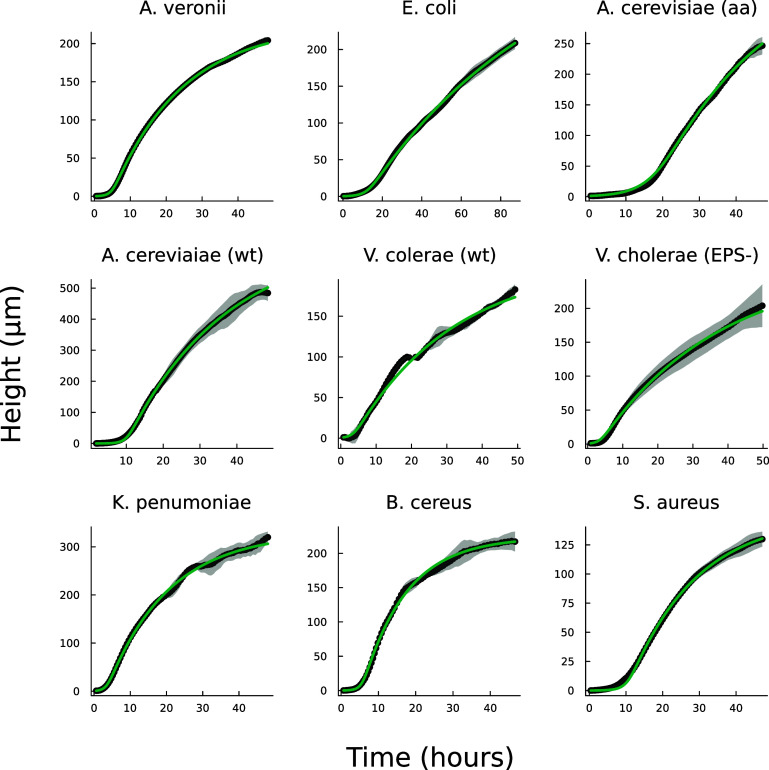
Displayed is the active fluid model fit to the same experimental data from Bravo *et al* [15] using equation (27). The fit is as precise as the interface model detailed in (1) with similar differences in fit parameters as shown in figure 1.

We now compare the interface model (as outlined in Bravo *et al* [15]) and the full analytical expression (equation (27)), referred to as the active fluid model, to experimental data (originally published in [15]). Figure 1(A) illustrates the best fits of both the interface and active fluid models against the height-over-time curve of an A. veronii colony (for this and all fitting in this paper, we use the first height recorded as the initial condition) , revealing that the two models are virtually indistinguishable. Furthermore, figure 1(B) highlights the similarity in form between the fluid and interface models, though the rationale behind the active fluid model’s form is more transparent, and it improves upon the interface model’s insights. To comprehensively demonstrate that the active fluid model is as robust as the interface model, we fitted the height-over-time curves of all nine strains outlined in [15], as presented in figure 3. As we observed previously, the fits between the two models are indistinguishable. This similarity is expected, given that both models are grounded in the same fundamental forms and share the same number of parameters. We also find that the fit parameters are all very similar to each other as seen in figure 1(A). This makes sense for *α* and *β*, as they represent growth and death, and both models treat growth and death similarly; we discuss *L* further below.

## 5. Resolving discrepancies in models of biofilm growth dynamics

**Figure 4.**
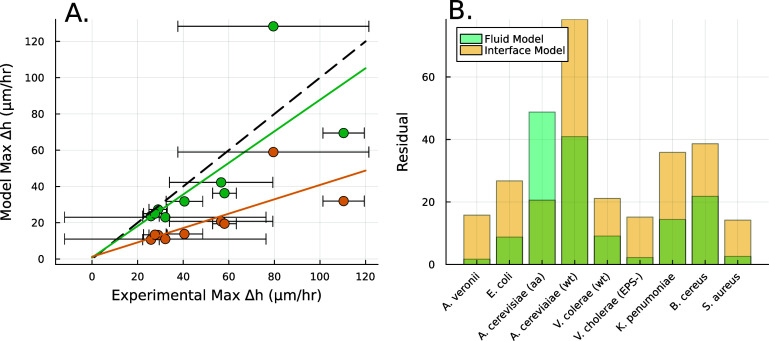
(A) The height $h^*$ at which $\Delta h / \Delta t$ is maximized, as calculated from equation (30), is plotted in green against the experimental peak in $\Delta h / \Delta t$ versus *h* from all data shown in figure 3. In orange, the *L* fit from the interface model is plotted against the same experimental peaks. (B) The residuals, representing the distance from the experimental peak in A and each model’s $h^*$, are displayed for each strain to illustrate how the active fluid model more accurately captures this phenomena in all but one strain.

Our objective is not to present a model that better fits the experimental data, but rather to validate that the broad functional form adopted is appropriate and captures a previously unaddressed observation. In particular, the interface model proposes a characteristic height, *L*, which marks the transition from the actively growing regime to the nutrient-depleted regime, a transition from mostly growth to mostly decay. This suggests that in every cellular layer above height *L*, the rate of decay exceeds the rate of growth. Thus, height *L* should represent the level at which biofilm growth reaches its maximum rate. However, experiments consistently show that the growth rate peaks (denoted $h^*$) at a biofilm height $h^* > L$; specifically, as illustrated in Figures 1(B) and 4(A), *L* does not align with $h^*$.

**Figure 5.**
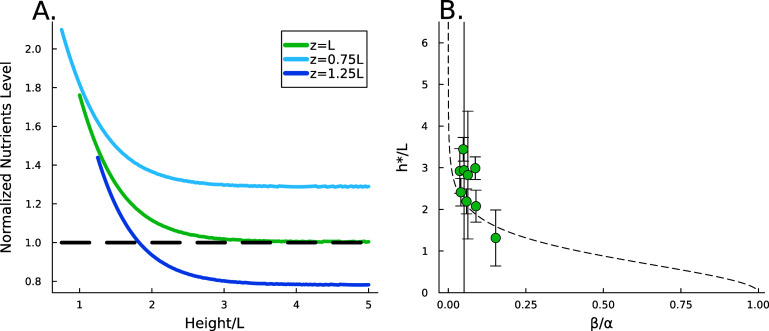
(A) The available nutrient concentration is shown at three different *z* distances as the height of the colony grows; we see that as the colony grows the nutrient concentration decreases. The black dashed line represents the steady state concentration at *z* = *L*, so we see that at low *h* the growing region is greater than *L*. (B) The ratio $h^*/L$ , where $h^*$ is the experimental height at which $\Delta h/\Delta t$ is maximized and *L* is the fit parameter from equation (27), is plotted against the fit parameters $\beta/\alpha$ as green circles. The dashed line represents equation (30). We observe that the strains from Bravo *et al* [15] occupy a closely aligned regime within the parameter space, and the active fluid model closely approximates the predicted $h^*/L$.

The active fluid model provides a framework to resolve this discrepancy. The best fit *L* values in both models are notably similar, which can be understood through the work presented in the previous section which shows that they measure the same phenomena. While *L* represents the characteristic length at which resources are depleted in both models, equation (28) shows that *L* only represents the size of the growing region when the colony achieves sufficient height ($h\gg L$); if $h\sim L$, then the thickness of the active growing region is greater than *L*. This phenomenon arises from the boundary condition on the upper surface of the colony where nutrients cannot flux outside of the biofilm; this prevents a simple characteristic decay of nutrients, as demonstrated by equations (21) and (22). The nutrients are ‘reflected’ back, so at low *h* the cells have an abundance of nutrients (see figures 5(A) and 2(B)). Thus, as the biofilm grows, the top surface becomes farther from the nutrient source (with increasing *h*), the taller biofilm develops a distinct characteristic depth *L* of metabolically active cells. This phenomenon is qualitatively illustrated in figure 2(A). Figure 5(A) demonstrates that when the colony initially attains height *L*, the nutrient concentration at *z* = *L* can be approximately 70% higher due to the boundary effect compared to later where *h* exceeds *L*, and the two converge. This interaction results in a more gradual transition in our final expression, and it facilitates the theoretical determination of $h^*$ through equation (30). Experimentally measured $h*$ values are much more aligned with the active fluid model predictions than with the interface model *L* (figures 4(A) and (B)).

An alternative explanation could be reached from figure 4(A) which demonstrates a nearly linear positive correlation between *L* and experimental $h^*$, this relationship might suggest there is residual growth beyond the characteristic nutrient decay length *L*, consistent with the persistent nature of exponential decay curves. In essence, *L* and $h^*$ may only be a factor apart, akin to one more, albeit smaller, standard deviation of nutrients. To explore this, we turn to the dimensionless ratio $h^*/L$, which represents the factor by which a colony’s maximum growth exceeds its characteristic length *L*. The active fluid model predicts this ratio via equation (30); it shows good agreement with experimental data, with seven of nine strains falling within one standard deviation of the active fluid model’s prediction (figure 5(C)). Furthermore, this analysis reveals that the examined strains occupy a narrow region in parameter space (specifically, $\beta / \alpha$). Thus, while the apparent proportionality between $h^*$ and *L* might suggest a universal relationship, this correlation may only hold when the ratio $\beta / \alpha$ remains relatively consistent across strains.

## 6. Discussion

This study presents a theoretical foundation for an empirically established model of vertical biofilm growth. Through rigorous mathematical analysis, we demonstrate that the heuristic ‘interface model’ previously developed in [15] emerges naturally from fundamental principles of active fluid dynamics.

Our analysis suggests that the *β* parameter in the heuristic model could primarily correspond to cell death with fast ‘breakdown’ of the dead cell structure. It is well established that bacteria exhibit characteristic death rates, and it is crucial to incorporate such dynamics in our models [37, 38]. Additionally, under conditions of nutrient scarcity (far from diffusing nutrients), bacteria have been observed to engage in programmed cell death [39]. In our model, the rate *β* is the the rate at which microbes die, and we assume it is substantially slower than the rate at which the residual volume of these cells is washed away or broken-down. This assumption simplifies the model by reducing the number of parameters, without significantly altering the dynamics under reasonable expectations.

To illustrate that this is a reasonable assumption, consider another bio-fluid, (*N*_2_), consisting of dead cells that are structurally intact, such that their volume still impacts the living bio-fluid *N*_1_. This material’s volume would increase at a rate $\beta N_1$ (due to death of living cells) and decrease at some rate *µ*, so $g_2^* = \beta N_1 - \mu N_2$. The breakdown rate *µ* encompasses all processes that remove the volume of non-growing material, including the diffusion of water originally within the cells out of the biofilm and the breakdown of organic material by other cells [40]. Given that the majority of cell volume is water, tracking the water from the dead bio-mass becomes the critical component to consider. Thus, similar to how nutrients diffuse at rates much faster than the biofilm growth rate, *µ* would be significantly higher than *β*. Therefore, under this fast ‘breakdown’ approximation, both the growth of *N*_2_ ($g_2^*$) and its relative value would be near zero, so $g_2^* = 0$ and $N_2 \ll N_1$. From this, we see that the system dynamics would remain essentially unchanged as the growth and volume of the dead mass is negligible. While additional factors,including viscous relaxation and extracellular matrix degradation, likely contribute to this term, we demonstrated here that cell death and lysis alone, under fast ‘breakdown’ conditions, can account for the observed *β* behavior. This finding elucidates why the relatively parsimonious heuristic model exhibits robustness across diverse microbial species [15].

We also validated our active fluid model against experimental data from the preceding study. The active fluid model demonstrates exceptional agreement in height-versus-time across nine distinct microbial strains. Furthermore, the model provides a theoretical framework explaining why growth rates achieve a maximum at heights exceeding the characteristic length *L*–a phenomenon that remained unexplained by the previous heuristic model. Our analysis reveals that boundary conditions at the colony’s upper surface enhance growth potential in shorter colonies. This insight suggests the possibility of analogous effects in horizontal growth at colony edges, where the cells are always exponentially growing and have two ‘no-flux’ bounds [13].

Intriguingly, the analysis presented here suggests that we observed a relatively small range of $h^*/L$ because the experiments happened to have a narrow range of $\beta / \alpha$. Future research could explore whether this narrow range of $\beta / \alpha$ was due to random chance, or if most microbes grown in the lab fall in this range. However, it is important to note that quantifying cell death rates within dense biofilm structures presents substantial experimental challenges.

An alternative approach to validating the active fluid model would involve extending the nutrient dynamics to two or three spatial dimensions as previous models typically assume logistic growth vertically. Concordance between such an extended model and experimental observations would provide compelling evidence for the model’s fundamental robustness, rather than mere consistency with existing data.
